# Development of an educational video on family planning for migrant women: a qualitative participatory co-design study in Northern Morocco

**DOI:** 10.1136/bmjph-2025-003458

**Published:** 2026-10-21

**Authors:** Mohamed Khalis, Oumnia Bouaddi, Maryam Hamidi, Hafida Charaka, Samira Harchaoui, Hanane Rassimi, Abdelhakim Yahyane, Radouane Belaouali

**Affiliations:** 1 Mohammed VI International School of Public Health, Mohammed VI University of Sciences and Health, Casablanca, Morocco; 2 Department of Public Health and Clinical Research, Mohammed VI Center for Research and Innovation, Rabat, Morocco; 3 Higher Institute of Nursing Professions and Health Techniques, Rabat, Morocco; 4 Hassan First University of Settat, Higher Institute of Health Sciences, Laboratory of Health Sciences and Technologies, Settat, Morocco; 5 Faculté de médecine et de pharmacie de Rabat, Université Mohammed V, Rabat, Morocco; 6 Ministry of Health and Social Protection, Rabat, Morocco; 7 Faculté de médecine et de pharmacie, University Hassan II, Casablanca, Morocco

**Keywords:** Public Health, Qualitative Research, Health Services Accessibility, Female, Community Participation

## Abstract

**Introduction:**

Migrant populations often face poorer sexual and reproductive health outcomes, including limited uptake of contraception. Morocco has become a country of transit and destination for thousands of sub-Saharan African (SSA) migrant women. Although these women are entitled to free contraception, they encounter informational barriers such as lack of knowledge about contraceptive options and limited access to trusted and culturally fit sources of information. Drawing on participatory research and design thinking approaches, this study aimed to co-design interventions to address informational barriers limiting family planning awareness and contraceptive use among SSA migrant women in Morocco.

**Methods:**

This participatory co-design study engaged 13 French-speaking SSA migrant women aged 18–49, recruited through a local non-governmental organisation (NGO) in Tangier, Morocco. A 1-day co-design workshop was conducted using the five-stage Design Thinking model: empathise, define, ideate, prototype and test. Participants used visual and interactive tools to propose, prioritise and shape educational interventions. The workshop session and post-feedback interviews were recorded, transcribed and analysed using rapid thematic analysis to identify intervention categories and themes.

**Results:**

Participants identified four approaches to address information gaps: culturally adapted educational materials, engagement of health professionals or community actors for direct counselling, outreach educational activities and embedding educational activities with vocational training or direct support activities (eg, provision of food items). They prioritised the first approach based on its feasibility, potential reach, sustainability and ability to directly engage users. Participants co-designed a short-animated video which was prototyped. The video included tailored content on contraceptive methods, information on entitlements to free services and pathways to family planning services. Design features were chosen such as relatable voiceover, skin tone representation, language and a preferred colour palette, in addition to preferred dissemination channels.

**Conclusions:**

This study shows that co-design is a feasible and meaningful method for developing culturally relevant educational interventions on contraception for migrant women. The next phase will focus on refining, implementing and testing the educational intervention, incorporating user feedback. There is also potential to adapt and expand the content to address a broader range of sexual and reproductive health needs in this community.

**WHAT IS ALREADY KNOWN ON THIS TOPIC**

- Sub-Saharan African migrant women in Morocco face significant informational barriers to accessing modern contraceptive methods, despite being entitled to free family planning services. Participatory research approaches are recognised for improving the relevance and equity of health interventions, particularly among underserved populations.

**WHAT THIS STUDY ADDS**

- This study shows the feasibility and value of using a participatory co-design approach with migrant women to develop culturally relevant educational interventions. It led to the creation and prototyping of a tailored animated educational video, with community-defined content, design features and delivery strategies.

**HOW THIS STUDY MIGHT AFFECT RESEARCH, PRACTICE OR POLICY**

- The findings support the integration of co-design and participatory approaches into sexual and reproductive health programming and intervention development for migrant communities in Morocco. This approach may improve the acceptability, build trust and promote uptake of services by aligning interventions with community preferences and experiences.

## Introduction

 Over the past decades, family planning (FP) has been recognised, beyond its positive health impact, as a key driver of women’s empowerment, improved educational attainment and expanded access to economic opportunities.^1,2^ Global commitment to FP peaked at the landmark International Conference on Population and Development in Cairo in 1994, which helped shape a global agenda to ensure access to FP services for all women.^3^ This commitment is further reflected in the Sustainable Development Goals, which include specific targets on universal access to sexual and reproductive health (SRH) services.^4^ Despite these global efforts, some populations, such as migrants and refugees, have historically been marginalised from accessing SRH services.^5,6^ Growing evidence shows that migrant women face disproportionate SRH risks due to multiple, overlapping vulnerabilities, including exposure to violence, precarious work conditions and poor access to services.^7^ Thus, they were shown to experience poor SRH outcomes compared with host population with several studies report higher rates of unintended pregnancies and abortions, inadequate prenatal care and low contraceptive coverage.^8–10^

In recent years, Morocco has seen an increase in the number of recorded migrant populations, reaching over 103 000 in 2020 according to the International Organization for Migration, largely from francophone Sub-Saharan African (SSA) countries.^11,12^ In response, the Moroccan government has led multisectoral efforts to improve migrant integration and ensure access to essential services, including healthcare. Several policy developments have extended entitlement to free primary and emergency care for migrants, along with access to selected vertical health programmes including tuberculosis, immunisation, HIV, hepatitis, FP, maternal and child health and reproductive healthcare.^13^ These programmes offer free services such as prenatal care, contraception, HIV testing and treatment and counselling. In addition, a network of NGOs collaborates closely with the Ministry of Health and Social Protection, as well as other stakeholders, to provide direct SRH services, particularly in the areas of HIV, peer-based accompaniment and contraception through community-based and mobile units. Despite these developments, localised and small-scale studies suggest that only around 20% of SSA migrants in Morocco report using any form of contraception.^14^ Specifically, the contraceptive prevalence rate among SSA women remains below the national average. A recent study in Rabat found that 37.9% of SSA women surveyed did not use contraceptives, and 11.2% reported having experienced unwanted pregnancies.^15^

This low uptake of contraception persists despite formal entitlements to services, suggesting that utilisation is constrained by persistent barriers. Qualitative studies among SSA women in two Moroccan cities highlight a lack of awareness about available SRH services, limited knowledge of rights and entitlements, and insufficient information to make informed decisions about their SRH.^16^ In the face of such informational barriers, tailored and context-specific solutions are needed to close the entitlement–utilisation gap and increase demand and uptake of contraception.

The role of participatory approaches in SRH intervention development for migrants is increasingly recognised in the global literature.^17–20^ A scoping review of SRH interventions among migrant populations found that coproduced interventions help build contextually sensitive educational content and improve self-empowerment and self-efficacy of migrant communities.^20^ Participatory research models have been shown to give voice to the lived experiences of participants and involve them as key partners in the research process, which has been shown to enhance the rigour, relevance and reach of science.^21^ These approaches are considered particularly valuable when working with underserved communities such as migrants, where power dynamics are often unequal and traditional top-down methods may fail to capture their voices or translate into interventions that communities trust and engage with.^22^ A systematic review by Rustage *et al* synthesising participatory approaches to developing health interventions for migrants found that, while such approaches embody core principles of inclusivity and democracy and show promise in generating contextually grounded interventions, the evidence base remains heavily concentrated in North America, with limited application in low- and middle-income countries.^22^ This geographic imbalance is particularly notable given that many of the most dynamic migration contexts, including South-South migration flows, are occurring in the Global South.

Digital health interventions (DHI) represent a growing and promising modality within the landscape of community-based SRH interventions.^17,23,24^ Evidence from a scoping review of DHIs developed for migrant and ethnic minority populations suggests that informational videos, mhealth tools and mobile-based platforms are increasingly used to deliver tailored health information to marginalised populations, given their low threshold for access and potential for wide reach.^24^ However, the same review found that the majority of DHIs are developed with minimal target group involvement as most studies evaluate acceptability or effectiveness only after the intervention has already been developed, without meaningful community input into content or design. Studies where target groups participated across all stages of development, from needs assessment through prototyping and evaluation, remain a small minority.

Morocco presents a relatively underexplored but important setting for this work. As a relatively new destination country embedded in a South-South migration context, it has made meaningful policy advances in extending health entitlements to migrants, particularly women. Research on migrant health in Morocco is an emerging and growing priority area, increasingly building on these policy developments. We argue it has reached a stage of maturity where the integration of participatory and community-led approaches into intervention development is both timely and necessary. Drawing on participatory research approaches and using the principles of co-design and design-thinking, this study aimed to develop interventions to address informational barriers to FP among SSA migrant women in Morocco.

## Methods

### Study design, setting

This participatory study with co-design principles aimed to co-develop a culturally tailored intervention with migrant communities in Tangier, Morocco, to strengthen their awareness and uptake of FP services. It was conducted by researchers in collaboration with a local migrant support organisation based in a neighbourhood where migrants live. Tangier is a city in northern Morocco that hosts a high number of migrants and is one of the most migrant-dense cities, mainly due to its proximity to Europe.

### Study participants and recruitment

Participants included 13 French-speaking SSA migrant women of reproductive age (18–49 years old) who participated in a 1-day co-design workshop and follow-up post-workshop feedback interviews. We chose this population as a target for the co-design study and subsequent intervention due to the fact that French-speaking migrants make up the majority in Morocco^11^ and this decision was also supported by the availability of literature showing low use of contraceptive methods among this group as well as a lack of information about the availability of and entitlement to free contraceptive methods and FP services.^16^ The group size of 13 was determined by practical considerations aimed at ensuring the workshop remained manageable and conducive to meaningful group discussion. The 1-day format was a pragmatic choice that allowed for focused, time-bounded collaborative feedback within the constraints of participant availability and organisational capacity. The 1-day workshop and post-workshop interviews were organised on 17 April 2025 at the premises of the branch of a Moroccan NGO in Tangier providing support to vulnerable populations including migrants who also supported participant recruitment. This study was funded by the International Development Research Centre, which covered all expenses.^25^ Participants received financial compensation in cash for transportation. They were also provided with lunch, coffee and refreshments during the workshop. As co-designing interventions is usually focused on localised interventions, the aim was not to achieve data saturation.

### Development of the intervention

This co-design project draws from a design thinking approach and methodology commonly used in health contexts. It uses an engaging problem-solving process that involves both staff and consumers to develop interventions and solutions to health challenges.^26^ This iterative process was proposed by Hasso Plattner Institute of Design at Stanford as a five-stage process which includes: building empathy to define a specific problem, ideating solutions, prototyping and testing (figure 1).^26^ The problem addressed in this research was identified through qualitative data collected as part of a broader project.^25^ This included a series of focus group discussions conducted in July 2024 with French-speaking migrants to explore barriers and facilitators to accessing SRH services. The findings (published elsewhere^16^) revealed multiple barriers, with one of the most prominent being the absence of reliable information on available SRH and FP services, and on how to access them. This research focuses mainly on the ideation and prototyping stages. It involves a workshop, during which migrant women brainstormed and co-designed interventions to address the above-mentioned informational barriers.

**Figure 1:**
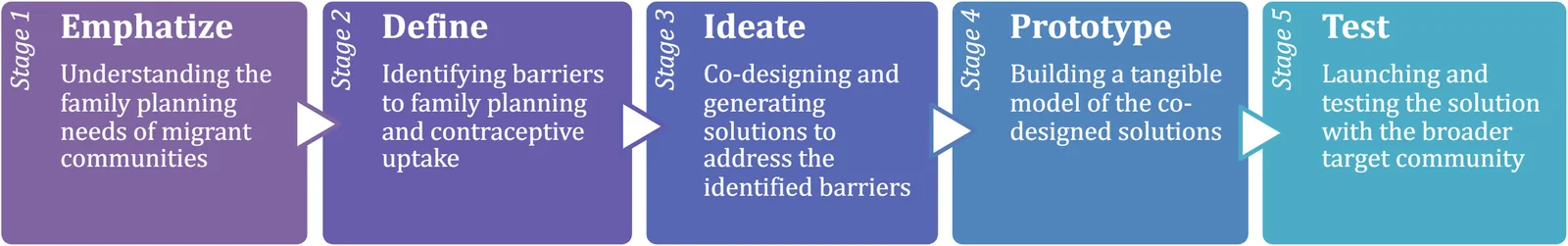
The design thinking process.

### Workshop organisation and activities

During the co-design workshop, participants were divided into two groups, each facilitated by two researchers with support from NGO community leaders. Prior to the workshop, the agenda was shared with the NGO for review and dissemination to prospective participants. Dedicated note-takers were assigned to each group. The session started with a project overview delivered by the project lead (MK), followed by icebreaker activities conducted by the researcher-facilitators. Then, participants were divided into two groups and provided with colourful pens, flipcharts and sticky notes. In the first stage, participants were guided to provide a list of all possible interventions to address information barriers to contraception. The facilitator recorded all ideas on the flipchart. In the second step, participants prioritised the identified ideas using a priority grid exercise. This exercise involved scoring the brainstormed interventions based on their potential effectiveness and feasibility. Each intervention was given a score of 0, 5, or 10 for each criterion, corresponding to low, moderate and high, respectively. For each intervention, the range of the score was from 0 to 20 (see online supplemental table S1 for priority grid in online supplemental data). The intervention with the highest score was retained and further developed using the ‘Four Ws’ framework (Who, What, When, How) to define intervention components. Participants were invited to think about where the intervention should be delivered, the specific target groups, the framing of the messaging and the content, the schedule and dissemination channels. All this information was recorded and participants were informed that a prototype will be developed and they will be solicited to provide feedback and suggest potential adjustments prior to testing. The resulting intervention design, derived from the workshop data, was presented to a representative from the Ministry of Health and Social Protection as part of implementation planning.

### Feedback on the co-design process

Following the workshop, individual feedback interviews were conducted to evaluate the co-design process, using a short interview guide adapted from existing literature on co-design methodologies (see online supplemental data).^27^

### Data analysis

The session and the post-workshop feedback interviews were recorded and notes and minutes from the workshop were compiled by members of the research team (HC and MH) and were subsequently transcribed. Rapid thematic analysis was conducted by two members of the research team using an inductive approach, allowing themes to emerge directly from participants’ feedback rather than being imposed by a predetermined framework. This was considered appropriate given the exploratory and iterative nature of the co-design process.

### Patient and public involvement

This study draws from participatory approaches that prioritise working with communities, ‘doing with’ rather than ‘doing to’ or ‘doing for’. Co-design workshops were held in familiar settings to build trust and ensure participants felt comfortable and empowered. To mitigate power imbalance and ensure voluntary participation, community leaders attended the sessions, providing participants with a familiar point of support. Researchers also drew on prior experience working with migrant communities in Morocco, and conducting activities in participants’ native language helped reduce language barriers. Importantly, participants in this study had decision-making power over the research outputs and the interventions developed. Their perspectives directly shaped the design, and their continued feedback will be actively sought during the prototype validation and testing stages.

## Results

### Sociodemographic data of participants

We included 13 migrant women aged between 21 and 41 years (mean: 29.7; SD: 6.4), originating mainly from Central Africa (Ivory Coast, Central African Republic, Senegal) (table 1). Duration of residence in Morocco was 4.3 years (SD: 2.4). The majority of participants were unemployed 9/13 while four reported having employment (in hairdressing, hospitality or self-employment). A total of 7/13 participants completed high school education whereas, 3/13 completed primary school and 2/13 had higher education degrees. Most women identified as migrants 11/13, with one refugee and one asylum seeker.

**Table 1:** General characteristics of participants

Variable	Distribution N (%)
Age in years, mean (SD)	29.7 (6.4)
Time of stay in years, mean (SD)	4.3 (2.4)
Education level	
Primary	3 (23.1)
Secondary	1 (7.7)
High school	7 (53.8)
Higher education	2 (15.4)
Vocational training	0 (0)
Employment status	
Employed	4 (30.8)
Unemployed	9 (69.2)
Country of origin	
Central African Republic	4 (30.8)
Ivory Coast	3 (23.1)
Senegal	2 (15.4)
Cameroon	1 (7.7)
Democratic Republic of the Congo	1 (7.7)
Guinea	1 (7.7)
Niger	1 (7.7)
Migrant status	
Refugee	1 (7.7)
Asylum seeker	1 (7.7)
Migrant	11 (84.6)
Number of children (mean)	1.7

### Approaches and interventions generated

Participants discussed several approaches to increasing knowledge of contraception and promoting demand and uptake (table 2) (step 1). The first overarching approach was the development of culturally tailored educational materials, including posters and flyers in health centres and community associations outlining FP methods and access points; short animated videos in French and English with voiceovers, and weekly online training materials shared via WhatsApp. The second approach included the engagement and training of health professionals and community actors (eg, staff from migrant women’s associations) to directly disseminate FP information through biweekly in-person awareness sessions in community associations each focused on a specific contraceptive method. The third approach focused on outreach awareness interventions including workshops in public parks. The fourth approach included embedding educational activities with the distribution of food items and baskets, and including FP messages into vocational training such as cooking or beauty workshops often organised at NGO premises.

**Table 2:** Approaches and intervention ideas generated

Approach	Generated intervention ideas
Development of educational materials	Posters in health centres and community associations explaining contraceptive methods and where to access them Educational flyers Short animated videos presenting family planning options and benefits
Awareness through engagement of health professionals and/or community actors	Training healthcare providers, community leaders, and women’s association staff to deliver family planning information Biweekly in-person sessions led by health professionals, each focusing on a specific contraceptive method Online training on family planning for migrants provided by health professionals
Outreach educational activities	Family planning awareness workshops held in public spaces (eg, parks)
Embedded educational activities	Distributing food baskets alongside family planning counselling and information sessions Integrating family planning education into vocational skills workshops (eg, cooking or beauty sessions)

Then, participants discussed the pros and cons of each intervention and conducted the prioritisation exercise (step 2). Participants highlighted that the involvement of intermediaries such as health professionals may be ‘counterproductive’ since the goal was to engage migrant women directly, thus, preference was given to direct interaction with educational content. The embedded approach was appreciated for its double benefit in offering both information and tangible support but was seen as limited in scale and sustainability due to resource constraints. Participants reached consensus that the development and dissemination of educational materials approach offered the best balance between feasibility and effectiveness. Educational materials such as flyers and posters were seen as static and requiring resources to be consistently invested in printing and disseminating in order to reach migrant women across different areas (in NGOs, health centres, community spaces, those living in shared accommodations ‘foyers’). Short animated educational videos were scored highest and participants described that they leverage digital spaces and have the potential to reach a large number of migrant women with a fixed cost or need for minimum adjustments over time. Thus, this intervention was taken to the next step of the co-design process to identify its various components.

### Description of the intervention: short animated educational video

Participants co-designed an animated video concept aimed at supporting informed FP choices. They preferred the use of animated characters with dark skin tones and the avoidance of real human faces. Participants highlighted that the video should cover key information on the importance of FP, how to access free contraceptive methods at primary health centres, and a clear explanation of available options (pills, implants, intrauterine devices (IUDs), injectables, condoms, etc). Participants recommended a length of 2 to 3 min to facilitate downloading and sharing on digital platforms, and feature a friendly, engaging narrator and a soft colour palette (violet, pink). Both French and English versions were recommended in order to reach anglophone SSA migrant women as well. Participants emphasised the importance of using minimal text to support audiences with limited literacy, and the use of simplified non-technical language. Participants mentioned that both men and women (15 years and above) should be targeted as the video should include both male and female contraceptive methods. Preferred dissemination channels included digital platforms frequently used by this population, including WhatsApp groups, Facebook community pages, TikTok, YouTube and channels of local associations and networks. Offline dissemination was also recommended through NGOs, hospitals, maternity wards, and during health promotion events such as Menstrual Hygiene Day. Preferred broadcasting times included evenings via WhatsApp and during daytime NGO activities and other events targeting migrant populations. This intervention was taken to the prototype stage.

### Prototyping

A freelance videographer was provided with a script co-developed by the research team and an FP expert (HR), along with guidance on tailoring the visual content to reflect participant preferences. During storyboard development, the lead authors requested adjustments to improve cultural appropriateness and viewer acceptance. These included dressing the midwife character in colours similar to those worn in Moroccan primary health facilities, and depicting migrant women in more modest clothing, such as long skirts, to align with the expectations of culturally conservative audiences. The storyboard is now finalised (see figure 2 for storyboard details), and the video is currently under development. The next steps will include feedback sessions to adjust and make user-informed changes with participants and the dissemination of the final video, followed by an evaluation of the intervention among a sample of migrant women.

**Figure 2:**
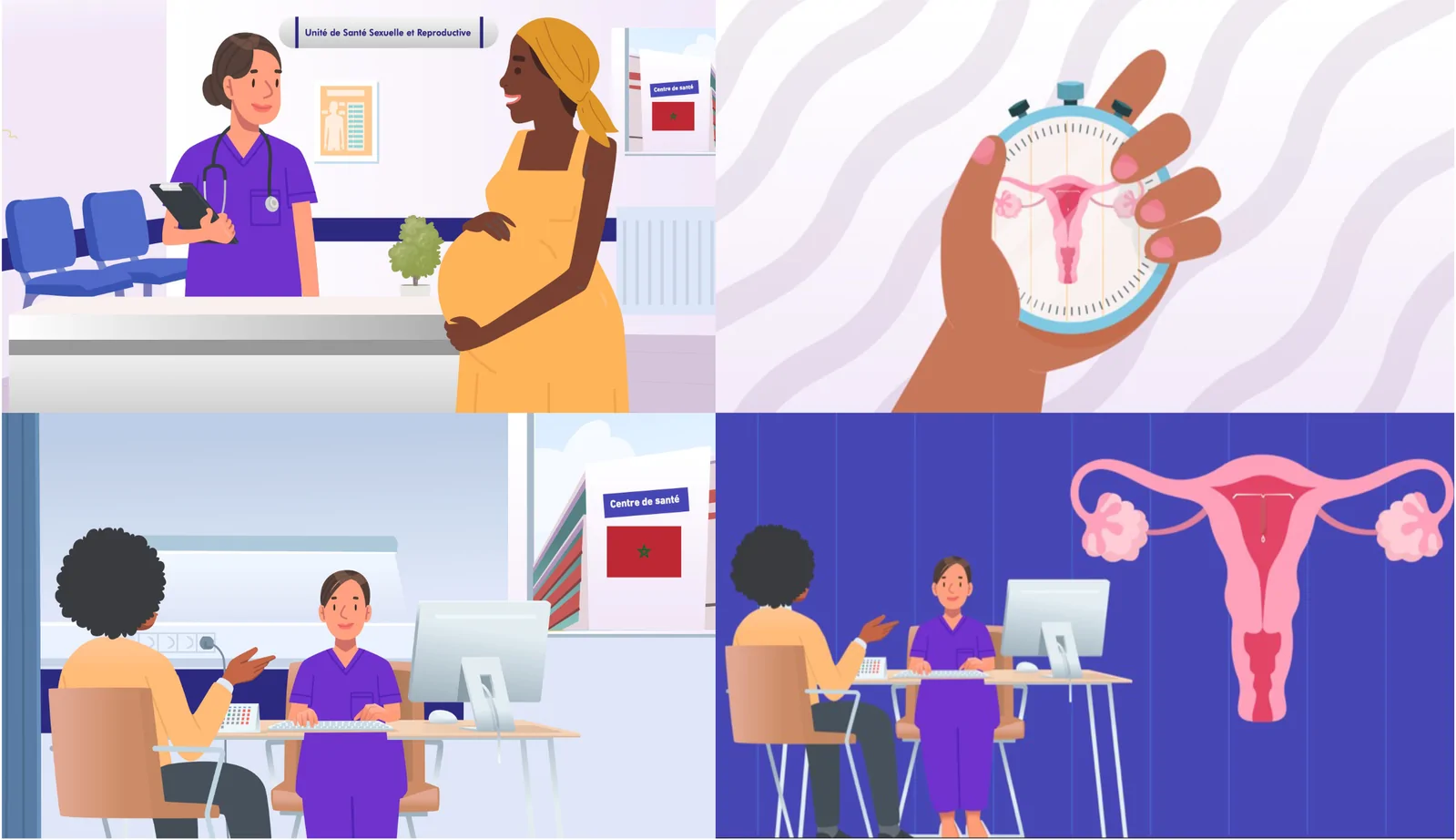
Storyboard captures from the prototype animated video.

### Feedback on the participatory process

The post-workshop feedback interviews revealed a high level of satisfaction with the co-design approach, which participants found engaging and empowering. All women reported active involvement in the workshop, contributing ideas, proposing solutions, participating in group discussions and helping to shape the content of tools such as the video and flyer. Participants did not experience themselves as passive informants but as genuine coauthors of the proposed interventions:

It’s a great idea to include us in creating solutions for our own lives – Participant

I felt integrated into the workshop, not just an observer – Participant

This sense of ownership was reinforced by a facilitation environment perceived as inclusive and respectful, where women felt heard and uninterrupted. The interactive format also deepened engagement, with participants emphasising the quality of collective exchange:

I was listened to, I spoke up and no one interrupted me – Participant

It was interactive, everyone was sharing – Participant

We had fun and we learned – Participant

This format also served an incidental educational function: several participants reported gaining new knowledge about FP during the workshop itself, including awareness that contraception services are free.

However, some challenges were raised, such as frustration over certain proposals being excluded due to limited resources, occasional group tensions and the lack of childcare services, which hindered participation for mothers:

Some ideas were not feasible, but I understood it was due to a lack of resources – Participant

## Discussion

The present study set out to co-design community-informed and context-specific interventions to address persistent informational barriers to contraception and FP among French-speaking SSA migrant women in Tangier, Morocco. The process resulted in the co-design of a short-animated educational video, to inform about entitlements to free-of-charge FP services, explain available contraceptive methods and outline practical steps for accessing services. Participants played a central role in shaping the content, language and design features of the video which was moved forward to the prototyping stage. The storyboard for the video has been finalised, and the final video is currently under development. We will make adjustments based on the same participants’ feedback prior to testing, disseminating the video and evaluating its impact on short-term and medium-term outcomes of interest (knowledge, uptake of contraception) in the broader target community. Feedback on the co-design process from participants suggested that they valued the opportunity to contribute, felt heard and appreciated being involved in developing solutions that directly affected their health.

The impact of education through educational materials chosen by participants in this study on improving modern contraceptive use is mixed. Systematic reviews examining the effects of such interventions have found a positive impact on contraceptive knowledge. For example, interventions such as interviews, counselling and interactive sessions, often involving provider–client interactions, significantly improved knowledge.^28^ However, the use of intermediaries was not preferred by participants in this study. This preference also aligns with some contrasting evidence showing that interactive communication supported by various health education methods such as brochures, booklets and peer educators was perceived as more effective than the one-way, routine counselling typically offered in FP education.^29^ Similarly, multiple studies specifically assessing educational tools, including decision aids, written materials, audio and video resources and interactive games, overall show a positive impact on contraceptive knowledge.^30,31^ However, there are limited robust studies measuring the direct impact on contraceptive behaviours, including actual uptake and sustained use^32^ with some research suggesting that knowledge may mediate other outcomes of interest.^33,34^ Nevertheless, it is worth noting that co-design approaches, because they are localised and grounded in community experiences, may lead to intervention strategies that diverge from traditional evidence-based approaches.^35^ However, co-design has been shown to strengthen the rigour and relevance of scientific research,^21^ including health interventions and may contribute to better outcomes by increasing acceptability, trust and contextual fit.^20^

Participants in this study expressed satisfaction with the co-design process and highlighted its role in empowerment and active participation. This is expected, as one of the issues and challenges in research among underserved groups such as migrants, reported in the literature, is fostering trust and building rapport^22^ and breaking the cycle of research fatigue that these groups often encounter.^36^ Often, participatory approaches in migrant health vary, with different levels of participant involvement across research stages.^37^ Participation of migrants through interviews and other data collection methods indirectly is good for generating evidence on existing issues, but it does not impact the distribution of power and equitability in research. The participatory co-design approach employed here proved valuable in both generating interventions but also in surfacing lived experiences and community-specific insights that would have likely remained hidden in a conventional top-down research process. Participants brought forward nuanced perspectives on what information mattered most to them, how it should be communicated and what cultural references or visual formats would be effective. The animated video, for instance, emerged from repeated participant feedback that visual storytelling would be more effective than printed materials for those with low literacy.

The strengths of this study include the use of design thinking tools and the demonstration of how this approach can be applied in real-world settings to benefit underserved migrant communities. The process resulted in an intervention co-created through participant interaction and lived experiences, thus ensuring relevance and cultural appropriateness. This study also has some limitations. The sample was limited to French-speaking SSA migrant women in Tangier, which may not capture the full diversity of migrant experiences across Morocco. Migrants are a heterogeneous group with diverse linguistic, cultural, and intersectional backgrounds. As co-design approaches are inherently oriented towards localised, context-specific interventions rather than broadly generalisable outcomes, data saturation was not a goal of this study, consistent with the aims of co-design methodologies more broadly.^22,38^ Nevertheless, we acknowledge that involving more diverse groups, as well as a larger number of participants, could have yielded richer and more varied input, potentially capturing perspectives from migrant women of different linguistic, national and cultural backgrounds beyond the French-speaking SSA population included in this study. Second, the assessment of the co-design process relied on tools and instruments available in the literature. The lack of standardised tools and structured evaluation frameworks may affect the comparability of findings across similar studies.^39^ Third, the co-designed interventions are still in the prototype or development stage. We have not yet assessed their effectiveness in improving knowledge, awareness or uptake of contraception. Despite these limitations, the study offers valuable insights into how participatory design can advance health equity and community empowerment for migrants particularly in settings where top-down health interventions often fail to reach or engage this group. A key limitation of this study relates to the boundaries of participant decision-making within the co-design process. While participants were actively involved in shaping the content and format of the educational tool, some ideas were excluded due to feasibility constraints, which necessarily limited the scope of their decision-making power. This reflects a common tension in participatory research between methodological ideals and practical realities.^22^ Furthermore, the educational tool developed in this study targets individual-level capabilities, specifically, increasing knowledge about contraception methods, rights and pathways to access among migrant women, rather than addressing the broader structural and systemic factors that contribute to informational barriers. We acknowledge that informational interventions of this kind may represent, in public health terms, a low-hanging fruit.^40^ However, tackling broader systemic issues would require wider stakeholder engagement efforts beyond the scope of this study. That said, the digital nature of the educational tool offers meaningful flexibility, as it can be readily integrated into routine educational activities delivered by civil society organisations working with migrant populations and by healthcare workers. Our partnership with the Ministry of Health and Social Protection provides a pathway to ensure broader dissemination and reach among actors involved in delivering FP education and services to migrant populations.

## Conclusions

This study showed that participatory co-design is both feasible and meaningful for developing culturally relevant educational material on family planning with migrant women in Morocco. The resulting animated video, co-designed to address informational barriers to FP among SSA migrant women in Tangier, holds potential for integration into existing health system and civil society efforts. Given that Morocco already offers free FP services through its primary healthcare network, the video could serve as a low-cost, scalable tool to generate demand and bridge the persistent gap between entitlement and utilisation. Next steps include participant feedback sessions to iteratively refine the prototype before testing and evaluating its impact on FP knowledge and service utilisation.

## Supplementary material

10.1136/bmjph-2025-003458 online supplemental file 1

## Data Availability

No data are available.
