# Supplementary material for: Development of an educational video on family planning for migrant women: a qualitative participatory co-design study in Northern Morocco
Source: BMJ Public Health. 2026 Oct 21;4(4):e003458. doi: 10.1136/bmjph-2025-003458 (PMC13691878; doi:10.1136/bmjph-2025-003458)
Supplement: Supplementary file 1 [file bmjph-4-4-s001.docx]

**Supplementary data**

**1. Table S1. Prioritization grid exercise**

| **List of interventions** | **Criteria** | | | | | | **Total (High effectiveness + high feasibility)** |
| --- | --- | --- | --- | --- | --- | --- | --- |
|  | **Effectiveness** | | | **Feasibility** | | |  |
|  | **Low (0)** | **Moderate (5)** | **High (10)** | **Low (0)** | **Moderate (5)** | **High (10)** |  |
|  |  |  |  |  |  |  |  |
|  |  |  |  |  |  |  |  |

**2. Post-workshop feedback interview guide**

1. How did you become involved in the co-design workshop? For example, what role did you come to the co-design workshop with?

2. Can you explain how you found the experience of participating?

- ○ Prompt. What was the best part? What was the least enjoyable part (if anything)?

3. Can you describe the extent you felt valued and supported being a part of the process? Prompt. Did you feel listened to and heard? If not valued why not?

4. What challenges do you think there were about the co-design process? What ways, if any were these challenges overcome?

5. Do you think the ideas you discussed in the co-design were listened to and taken on board? Elaborate on why or why not

6. If anything should or could be done differently in the future what would that be and why? Prompt. What lessons could be learnt?

7. Is there anything else you would like to tell me about your participation in the co-design workshop?
